# Retraction: Verheijen, B.M. Expression Profile of Long Non-Coding RNAs during Early Postnatal Development of Mouse Spinal Cord. *Non-Coding RNA* 2020, *6*, 18

**DOI:** 10.3390/ncrna6030031

**Published:** 2020-08-10

**Authors:** Non-Coding RNA Editorial Office

**Affiliations:** MDPI, St. Alban-Anlage 66, 4052 Basel, Switzerland; ncrna@mdpi.com

The published article [1] has been retracted at the request of Utrecht University owing to several issues with the article (lack of reference to funding sources, no consent for publication from the supervisor, no mention of co-authors) and it has been removed due to legal issues of data confidentiality. Dr. Verheijen agreed that this version of the manuscript should be retracted to allow further scrutiny of the presented data. Thus, out of respect for the institution’s wishes, in agreement with the *Non-Coding RNA* Editorial Office, and in agreement with the author, the paper will be removed from the public record and marked as retracted. We apologize for any inconvenience caused by the removal of this article.

MDPI is a member of the Committee on Publication Ethics (COPE) and takes very seriously the responsibility to enforce strict ethical policies and standards. The article [1] is retracted and shall be marked accordingly.
